# Physical and cultural determinants of postpartum pelvic floor support and symptoms following vaginal delivery: a protocol for a mixed-methods prospective cohort study

**DOI:** 10.1136/bmjopen-2016-014252

**Published:** 2017-01-10

**Authors:** Ingrid E Nygaard, Erin Clark, Lauren Clark, Marlene J Egger, Robert Hitchcock, Yvonne Hsu, Peggy Norton, Ana Sanchez-Birkhead, Janet Shaw, Xiaoming Sheng, Michael Varner

**Affiliations:** 1 Department of Obstetrics and Gynecology, School of Medicine, University of Utah, Salt Lake City, Utah, USA; 2 College of Nursing, University of Utah, Salt Lake City, Utah, USA; 3 Department of Family and Preventive Medicine, School of Medicine, University of Utah, Salt Lake City, Utah, USA; 4 Department of Bioengineering, College of Engineering, University of Utah, Salt Lake City, Utah, USA; 5 Department of Health, Kinesiology, and Recreation, College of Health, University of Utah, Salt Lake City, Utah, USA; 6 Department of Pediatrics, School of Medicine, University of Utah, Salt Lake City, Utah, USA

**Keywords:** pelvic floor disorders, physical activity, intra-abdominal pressure, urinary incontinence, Pelvic organ prolapse, childbirth injury

## Abstract

**Introduction:**

Pelvic floor disorders (PFDs), including pelvic organ prolapse (POP), stress and urgency urinary incontinence, and faecal incontinence, are common and arise from loss of pelvic support. Although severe disease often does not occur until women become older, pregnancy and childbirth are major risk factors for PFDs, especially POP. We understand little about modifiable factors that impact pelvic floor function recovery after vaginal birth. This National Institutes of Health (NIH)-funded Program Project, ‘Bridging physical and cultural determinants of postpartum pelvic floor support and symptoms following vaginal delivery’, uses mixed-methods research to study the influences of intra-abdominal pressure, physical activity, body habitus and muscle fitness on pelvic floor support and symptoms as well as the cultural context in which women experience those changes.

**Methods and analysis:**

Using quantitative methods, we will evaluate whether pelvic floor support and symptoms 1 year after the first vaginal delivery are affected by biologically plausible factors that may impact muscle, nerve and connective tissue healing during recovery (first 8 weeks postpartum) and strengthening (remainder of the first postpartum year). Using qualitative methods, we will examine cultural aspects of perceptions, explanations of changes in pelvic floor support, and actions taken by Mexican-American and Euro-American primipara, emphasising early changes after childbirth. We will summarise project results in a resource toolkit that will enhance opportunities for dialogue between women, their families and providers, and across lay and medical discourses. We anticipate enrolling up to 1530 nulliparous women into the prospective cohort study during the third trimester, following those who deliver vaginally 1 year postpartum. Participants will be drawn from this cohort to meet the project's aims.

**Ethics and dissemination:**

The University of Utah and Intermountain Healthcare Institutional Review Boards approved this study. Data are stored in a secure password-protected database. Papers summarising the primary results and ancillary analyses will be published in peer-reviewed journals.

**Strengths and limitations of this study**

- We objectively assess physical activity using accelerometry and intra-abdominal pressure using a vaginal transducer system developed by a collaboration among our bioengineering, exercise science and urogynaecology researchers.
- Intrapartum events are systematically collected to enable stratification and adjustment for these risk factors.
- The current protocol does not assess levator ani muscle injury.
- This study does not include long-term follow-up of participants, but does establish a registry to enable such an effort in the future.

## Introduction

Pelvic floor disorders (PFDs) are common.^1^ Up to one in seven women have surgery for pelvic organ prolapse (POP) or urinary incontinence (UI) in their lifetime.^2–4^ In the USA, the direct cost of treating these disorders exceeds $1 billion per year.^5–7^ As the population ages, the number of women suffering from PFDs is expected to increase, resulting in a large social, medical and economic burden.^8^ It is surprising how little we understand about the modifiable factors that contribute to these disorders, in particular POP, despite the huge burden on women and the healthcare budget associated with these disorders. The focus of most existing research has been in women presenting for treatment but not on prevention.

Changes in pelvic floor support are experienced by women across the lifespan. Pregnancy and childbirth are major risk factors for PFDs, though severe disease often does not manifest itself until women become older.^9–17^ POP is almost entirely an effect of vaginal delivery, parity and time since delivery.^18^ ^19^ After one vaginal delivery, a quarter to half of the women demonstrate a mild prolapse during the first postpartum year, while half report urinary and 17% report faecal incontinence.^20–22^ Young women demonstrate a range of pelvic floor support and pelvic floor symptoms, which may affect quality of life and sexual activity.^20^ ^23–32^ If symptoms become persistent and bothersome, and are accompanied, depending on the condition, by objective findings, they are considered PFDs, most commonly POP, stress UI (SUI) and faecal incontinence.^1^ ^33^

Other than vaginal birth, few modifiable risk factors for POP, including obesity and heavy lifting, have been identified.^34–38^ Scant data suggest that women with POP are more likely to report a history of strenuous jobs than women without.^39–43^ Constipation, which similar to strenuous work increases intra-abdominal pressure (IAP), is inconsistently associated with POP.^36^ ^44^ ^45^

**Figure 1:**
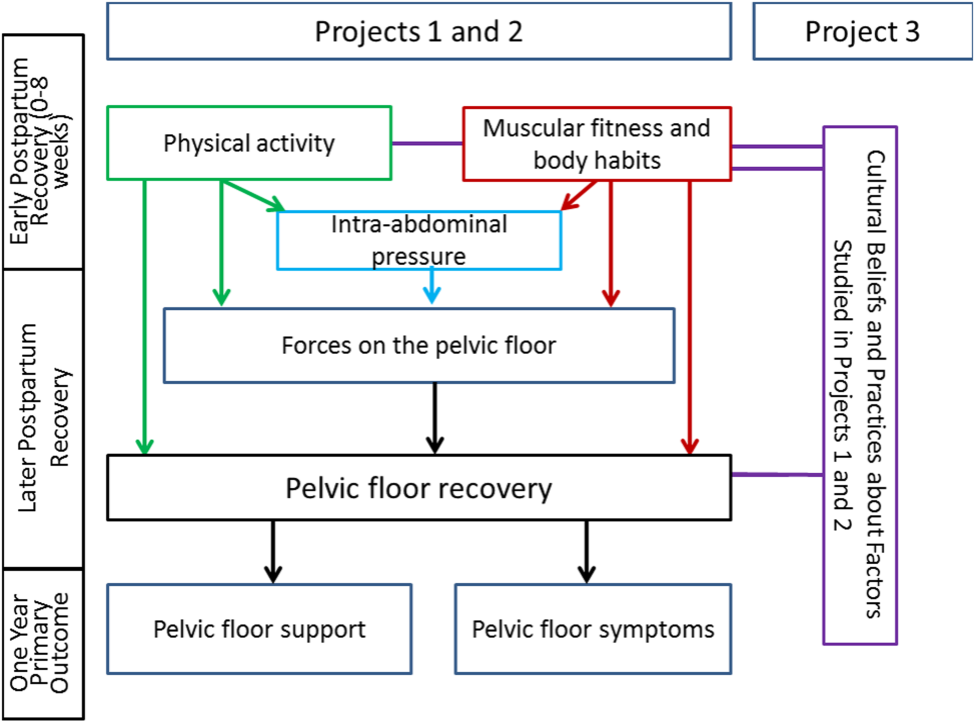
Inter-relationships between the three projects in this Program.

Vaginal delivery affects pelvic muscles, nerves and connective tissue, which clinically may be seen as loss of pelvic floor support. Over the past decade, we have gained important information about some of the ways in which vaginal delivery affects the structure and function of the pelvic floor.^46–48^ However, we know very little about how pelvic floor function recovers after vaginal delivery. In this Program Project, summarised in figure 1, we will study whether the postpregnancy milieu, including physical and cultural factors, add to the effects of vaginal childbirth on the pathogenesis of PFDs. In two quantitative projects, we will evaluate whether pelvic floor support and symptoms 1 year after the first vaginal delivery are affected by biologically plausible factors that may impact muscle, nerve and connective tissue healing during the postpartum recovery period (first 8 weeks postpartum) and pelvic floor function during the postpartum strengthening period (remainder of the first postpartum year). Specifically, we will evaluate the timing and dose of moderate/vigorous physical activity and inactivity, and timing of and exposure to a range of IAPs. Finding relationships between physical activity, indices of muscular fitness, body habitus, IAP and pelvic floor support or symptoms will provide realistic targets for disease prevention and pelvic floor health management. In a qualitative project, we will examine the cultural aspects of perceptions, explanations of pelvic floor support changes and actions taken by Mexican-American and Euro-American primiparas, emphasising early changes after childbirth. Summarising the projects' results in a resource toolkit will enhance opportunities for dialogue between women, their families and providers, and across lay and medical discourses, with a view towards workable prevention strategies.

## Methods and analysis

### Study overview

This National Institutes of Health (NIH)-funded Program Project, ‘Bridging physical and cultural determinants of postpartum pelvic floor support and symptoms following vaginal delivery’ (abbreviated as *M*otherhood *A*nd *P*elvic health (MAP)), encompasses a prospective cohort study and a qualitative study and will enrol ∼1530 nulliparous women during the third trimester. After excluding women who subsequently deliver by caesarean section, deliver preterm (<37 weeks gestation), are pregnant at 1 year postpartum and after accounting for study withdrawals, we will evaluate the remaining estimated 585 women at 1 year. These women make up the primiparous cohort from which participants are drawn to meet the objectives of the Program. We will measure:

- Pelvic floor support and symptoms at the third trimester, and at 8 weeks and 1 year postpartum;
- Antepartum predictors in the third trimester;
- Delivery risk factors following vaginal birth;
- Physical activity predictors via accelerometry at 2–3 and 5–6 weeks and 6 months postpartum;
- IAP, abdominal muscle endurance and waist circumference 8 weeks and 1 year postpartum; and
- Muscular fitness and body composition 1 year postpartum.

We chose to define the acute postpartum recovery period as 8 weeks both because of the biological plausibility noted above and because in the USA, 6–10 weeks coincides with the postpartum clinical assessment, aiding the feasibility of our study, and many working women would have returned to work by this time point.

Thirty Euro-American and 30 Mexican-American participants will be purposefully selected for qualitative interviews following the 8-week and 1-year visits. Women will be offered participation in a Pregnancy and Pelvic Floor Outcomes Registry, to be established with this study population.

This study is registered in clinicaltrials.gov (NCT02512016).

### Setting

The study will be conducted in Salt Lake City, Utah at the University of Utah Hospitals and Clinics and LDS Hospital (part of Intermountain Healthcare) and at additional clinic sites planned to maximise enrolment of Hispanic women, including Community Health Centers. Planning for this study was finalised after notification of funding in July 2015; the projected study end date is 30 June 2020.

### Participants

Participants will be ≥18 years, English or Spanish speaking, nulliparous with a singleton gestation, ≥28 weeks gestation, planning vaginal delivery, and not planning to move to a location precluding follow-up. Women will be excluded if they are unable to walk independently before pregnancy without aids, or if they have major medical or surgical problems precluding physical activity for the past 12 months; have conditions such as Marfan's or Ehlers-Danlos which may increase the risk of POP; were treated surgically for POP or UI before pregnancy; or do not have email, cell phone or landline telephone access. While women with obstetric complications, such as stillbirth or unanticipated poor neonatal outcome, are not excluded from further study participation after delivery, they are sensitively offered the opportunity to withdraw from the study or continue, as per their preference.

Participants for the qualitative study will be purposefully selected, primarily from the participant pool above. Additional inclusion criteria for the qualitative study are women who self-identify as non-Hispanic white Americans of European descent and report at least second-generation US residence; or self-identify as Latina, Hispanic, Mexican-American, or American of Mexican descent, of any race, and claim heritage from Mexico. These represent the two largest ethnic groups in Utah.

### Screening and recruitment

At each recruitment site, certified research screening staff will review all upcoming prenatal clinics, the electronic medical records and report potentially eligible women to the MAP study coordinators. MAP coordinators, fluent in English and/or Spanish, will then confirm potential eligibility based on chart review and face-to-face interview during a clinic visit. Depending on clinic flow, the consent process and initial study procedures will occur in the same setting as the prenatal visit or in a research examination room. We will report the numbers of women potentially eligible, evaluated for eligibility, confirmed eligible and included in the study. Investigators for the qualitative research study will use data from participants' 8-week and 1-year postpartum visits to select participants, using iterative, purposive sampling.

### Outcome measures, variables, measurement and data sources for quantitative study aims

The timing and method of obtaining outcome and explanatory variables are summarised in table 1. Additional information about key variables follows.

**Table 1:** Timing and method of obtaining outcome and explanatory variables

Category	Explanatory variable	Method	Time*
Physical attributes	Weight, height	Calibrated scale; wall stadiometer	V1, V2, V3
	Muscular fitness	AME	V2, V3
		Pelvic muscle strength	V3
		Grip strength	V3
	Body habitus	Waist circumference	V2, V3
		Air displacement plethysmography (BodPod)	V3
	Pre-pregnancy weight	Self-report	V1
Medical history	Chronic cough	As per NHANES†	V1, V2, V3
	Constipation	Defecation distress	V1, V2, V3
	Medical conditions	Inventory	V1, V2, V3
	Health status	Checklist	V1, V2, V3
	Postpartum wound	1-item questionnaire	V2
	Breastfeeding status	Self-report	V2, V3
	Hormonal contraception	Self-report	V2, V3
	Pre-pregnancy recurrent	Self-report	V1
	Urinary tract infection	Self-report	V1, V2, V3
	Pelvic floor muscle exercise	Self-report	V1, V2, V3
Demographic information	Age	Date of birth	V1
	Race/ethnicity	Self-report	V1
	Education	Self-report	V1
Demographic information for the multiple cohort		Self-report	V1
Cultural background	Acculturation index for self-identified Mexican women	SASH	V1
Delivery information	High-risk/other delivery variables‡	Chart abstraction	Delivery
Postpartum practices	Specific practices	Question checklist	V2
PA	MVPA, min/day	Accelerometry	2–3 and 5–6 weeks and 6 months pp
	Light intensity PA, min/day	Accelerometry	
	Moderate intensity PA, min/day	Accelerometry	
	Vigorous intensity PA, min/day	Accelerometry	
	Activity bouts of MVPA, min/day	Accelerometry	
	Current types of activity	Self-report	V1, 2–3 weeks pp, V2, 6 months pp, V3
	Pre-pregnancy PA level	Checklist based on BLHQ	V1
	Current PA level	RAPA RAPA	V1, 2–3 weeks pp, V2, 6 months pp, V3
IAP	IAP during AME	Vaginal sensor	V2, V3
	IAP during lift 12.5 kg	Vaginal sensor	V2, V3
Inactivity time	Hours/day	Accelerometry Self-report, as per NHANES†	6 months pp V1, V2, V3
Connective tissue fragility, linked to POP	Easy bruisability	Questionnaire	V1, V2, V3
	Varicose veins	Questionnaire	V1, V2, V3
	Stretch marks	Questionnaire	V1, V2, V3
Lifestyle factors linked to continence	Caffeine intake	Questionnaire	V1, V2, V3
	Tobacco	Questionnaire	V1, V2, V3
	Pelvic muscle exercises	Self-report	V1, V2, V3
Pelvic floor symptoms	Symptom with bother ≥2 domains	Self-administered EPIQ§	V1, V2, V3
	Stress urinary incontinence; overactive bladder, anal incontinence	EPIQ	V1, V2, V3
	Defaecation dysfunction	Defecation Distress Inventory	V1, V2, V3
	Pre-pregnancy urinary incontinence	Incontinence Severity Index, recall	V1
	Incontinence severity	Incontinence Severity Index	V1, V2, V3
Pelvic floor support	Maximum vaginal descent	Pelvic Organ Prolapse Quantification examination	V1, V2, V3
PFM function	PFM strength	Brinks scale Force using instrumented speculum	V1, V2, V3 V3
Women's experiences	Qualitative interview	Interviewer-administered	V2, V3

*V, visit; V1, third trimester; V2, 8 weeks; V3, 12 months; pp, postpartum.

†NHANES, National Health and Nutrition Examination Survey.

‡High-risk delivery variable: second stage labour >120 min, forceps, anal sphincter tear or shoulder dystocia. Additional delivery variables: birth weight, head circumference, rate of first stage (cm dilation/time), vacuum delivery, epidural.

§EPIQ, Epidemiology of Prolapse and Incontinence Questionnaire. Domains include defaecatory dysfunction, stress urinary incontinence, prolapse, overactive bladder, pain and difficult voiding, and anal incontinence.

AME, abdominal muscle endurance; BLHQ, Bone Loading History Questionnaire; IAP, intra-abdominal pressure; MVPA, moderate-to-vigorous physical activity; PA, physical activity; PFM, pelvic floor muscle; POP, pelvic organ prolapse; RAPA, Rapid Assessment of Physical Activity; SASH, Short Acculturation Scale for Hispanics.

#### Outcomes

We will capture pelvic floor health 1 year postpartum with two primary outcomes: pelvic floor support and pelvic floor symptoms. The first, an anatomic outcome, will be assessed using the Pelvic Organ Prolapse Quantification (POP-Q) system,^49^ a reproducible method for assessing vaginal descent.^50^ ^51^ The lowest level of vaginal descent during Valsalva is measured relative to its distance in centimetres (cm) from the hymen; points above the hymen are negative and those below are positive; vaginal support at the level of hymen is represented as ‘0 cm’. Maximum vaginal descent (MVD) represents the greatest observed descent of the anterior, posterior or apical vagina. Pelvic floor support will be categorised into MVD above the hymen (<0 cm; ie, better support) versus at or below the hymen (≥0 cm; worse support). This cut-point is commonly used in research because it represents the level at which more women become symptomatic.^52^ ^53^ Pelvic floor symptoms will be assessed using the Epidemiology of Prolapse and Incontinence Questionnaire (EPIQ),^54^ which contains 22 stem questions related to pelvic floor symptoms, and was validated in a diverse population of women seeking, as well as not seeking, care. To represent symptom burden, the symptom outcome will be dichotomised as the presence of symptoms accompanied by at least minimal bother (>0 on the visual analogue scale answered by women who endorse a symptom) in at least 2 domains on the EPIQ versus 0 or 1 domain.

Secondary 1-year outcomes include the symptoms of SUI and of an overactive bladder, both assessed using the EPIQ, UI severity, assessed using the Incontinence Severity Index^55^ ^56^ and constipation, assessed using the two constipation items from the Defecation Distress Inventory.^57^ ^58^

We chose to measure these outcomes also during the third trimester to enable us to account for predelivery pelvic floor health in our analyses.

#### Exposures

*Physical activity*: We will use accelerometers to measure moderate-to-vigorous physical activity and inactivity using established algorithms for our primary analyses, which objectively and reliably assess these constructs in a variety of populations, including pregnant and postpartum women.^59^ ^60^ To improve compliance, and consistent with the current National Health and Nutrition Examination Study (NHANES) 2011+ protocol, we chose wrist-worn accelerometers, the accuracy of which has been validated against waist-worn ones.^61–65^ The water-resistant triaxial Actigraph GT3X+ monitor accelerometer was highly associated with energy expenditure determined from indirect calorimetry in adults, predicted activity type with >82% accuracy, and had good correlation for concurrent associations between the wrist-worn GT3X+ and ground reaction forces in adults.^66–69^

Our participants will be instructed to wear the accelerometer on the non-dominant wrist continuously (24 hours) for 7 days at 2–3 and 5–6 weeks postpartum and again at 6 months postpartum. The GT9X looks like a wristwatch, has an liquid crystal display with current time but no feedback will be provided to participants regarding activity, and its internal mechanism for detecting body acceleration is identical to the earlier ActiGraph model (GT3X+) used in NHANES surveillance of population physical activity.

To provide additional descriptive data, participants will complete the Rapid Assessment of Physical Activity questionnaire,^70^ validated in English and Spanish, as well as a checklist based on the Bone Loading History Questionnaire^71^ summarising types of activities performed, during the third trimester, and at 2–3, 8 weeks, 6 months and 1 year postpartum. At the baseline questionnaire in the third trimester, they will use these instruments to report on physical activity level and type in the year before pregnancy.

*Intra-abdominal pressure*: We will use a vaginal sensor system developed by our group^72^ ^73^ to measure IAP at 8 weeks and 1 year postpartum. With the sensor in place, participants will complete a test of abdominal muscle endurance (described below) and will lift a weighted baby car seat weighing 12.5 kg (5.7 kg baby+6.8 kg car seat) three times. Since a prolapse tends to be less severe in young women than in women seeking treatment, it is unlikely that the sensor will become dislodged by a large vaginal bulge. Participant pressure data will be continuously recorded during each activity. Using our data converter software, we will calculate the primary predictor, mean maximal IAP, as well as other measures including mean IAP, area under the curve and first moment of the area.^74^

- Muscular fitness: since one composite measure of muscular fitness does not exist, we chose measures that are hierarchically associated with our primary outcomes of pelvic floor support and symptoms. The most specific measure of fitness in the pelvic floor is pelvic floor muscle strength. Fitness of the trunk musculature is important in the development of IAP. Finally, upper body muscle strength will be approximated using grip strength of the dominant hand.
- Pelvic floor muscle strength: measured using a valid and reliable instrumented speculum designed to minimise the effect of IAP on pelvic floor muscle strength.^75^
- Abdominal muscle endurance: measured once using a standard, reliable protocol^76^ and recorded as a maximal hold time (seconds; owing to participant fatigue, further repetitions are not possible).
- Grip strength: tested using the dominant hand, expressed as kg of force using a standard hydraulic hand dynamometer.^77^

*Body habitus*: We will measure both body mass index (BMI) and waist circumference, both of which correlate with certain pelvic floor outcomes, as well as body composition, which has not yet been explored in PFDs.^78–80^

- Waist circumference: determined by the mean of two measures (cm) at the natural waist.^81^
- BMI, kg/m^2^: weight (kg) and height (m) on a calibrated scale and wall stadiometer.
- Body composition (expressed as % fat mass): determined using air displacement plethysmography (BodPod, COSMED); this has acceptable validity and reliability when compared with hydrodensitometry.^82^

### Procedures and data sources for the qualitative study aims

This is a focused comparative ethnography study using guided, unstructured individual interviews as the primary data collection method to elicit women's personal narrative experience and discourse about sociocultural understandings of pelvic floor changes.^83^ The initial grand tour question asks women to describe their recovery since childbirth and changes they may have noted in the pelvic area, with follow-up questions and prompts. A purposefully selected sensitising sample of 8 Mexican-American (MA) and 8 Euro-American (EA) women with a symptomatic prolapse recruited from clinics will be interviewed first, followed by 30 MA and 30 EA new mothers from the MAP cohort. The sensitising sample will familiarise researchers with the culturally situated experiential trajectory of prolapse. Interviews will be conducted by qualified and trained research staff in the language chosen by the woman (English or Spanish) at a site she identifies as comfortable and private. Interviews will be recorded, transcribed in the original language, and then translated into English by a professional bilingual transcription service and verified by bilingual/bicultural research team members, adherent to Health Insurance Portability and Accountability Act (HIPAA) regulations.^84^ Analysis will follow standard ethnographic research practices.^85^ The ethnographic account will elicit detailed descriptions of the experience, and investigators will intensively compare across accounts, identify cultural interpretations of prolapse symptoms and pelvic floor changes, and refine conceptual categories to clarify the theoretical contribution of the ethnographic research analysis.^86^

### Study aims and adequacy of sample size for quantitative projects

The aims for the quantitative projects, as well as additional information related to sample size calculations where relevant, are summarised in table 2.

**Table 2:** Quantitative study aims, hypotheses and sample size considerations

Aim: to determine…	Hypotheses	Two-sided significance level	Minimal detectable OR
Whether IAP measured at 8 weeks postpartum during (a) lifting and (b) abdominal muscle endurance testing predicts pelvic floor support and symptoms 1 year postpartum.	(a) Higher IAP at 8 weeks postpartum during (a) lifting and (b) abdominal muscle endurance testing predicts worse pelvic floor support 1 year postpartum.(b) Higher IAP at 8 weeks postpartum during (a) lifting and (b) abdominal muscle endurance testing predicts greater pelvic floor symptoms 1 year postpartum.*	0.025	1.78
Whether measures of muscular fitness modify the effect of IAP during lifting on pelvic floor support at 1 year postpartum.	Women with high IAP during lifting 1 year postpartum who also demonstrate lower abdominal muscle endurance, less pelvic floor muscle strength or less grip strength 1 year postpartum will have higher odds of worse pelvic floor support at 1 year postpartum than women with high IAP but greater muscular fitness, whereas women demonstrating low IAP will have more similar odds of worse pelvic floor support regardless of fitness.†	0.017	1.82
Whether MVPA in the early postpartum period predicts pelvic floor support and symptoms 1 year postpartum.	(a) Greater daily average MVPA in the early postpartum period, measured using accelerometry at 2–3 and 5–6 weeks postpartum, predicts worse pelvic floor support 1 year postpartum.(b) Greater daily average MVPA in the early postpartum period, measured using accelerometry at 2–3 and 5–6 weeks postpartum, predicts greater symptoms 1 year postpartum.	0.05	1.70
Whether sedentary time during the later postpartum period, independent of MVPA, predicts pelvic floor support 1 year postpartum.	Greater daily average sedentary time measured using accelerometry for 7 days at 6 months postpartum is associated with worse pelvic floor support, independent of MVPA measured during the same time period.	0.05	1.70
Whether the presence of a high-risk delivery variable (forceps, prolonged second stage of labour, shoulder dystocia, anal sphincter laceration) modifies the association between MVPA in the early postpartum period on pelvic floor support and symptoms at 1 year (exploratory aim).	(a) The prevalence risk of worse pelvic floor support at 1 year will be higher for women with greater MVPA in the early postpartum period, higher in women with a high-risk delivery variable; and even higher for women with both.(b) The prevalence risk of greater pelvic floor symptoms at 1 year will be higher for women with greater MVPA in the early postpartum period, higher in women with a high-risk delivery variable; and even higher for women with both.	0.05	1.70
Whether each of grip strength and abdominal muscle endurance is associated with pelvic floor support and symptoms, independent of PFM, all measured at 1 year.	(a) Greater grip strength and greater abdominal muscle endurance are each associated with better pelvic floor support, adjusted for PFM strength.(b) Greater grip strength and greater abdominal muscle endurance are each associated with fewer symptoms, adjusted for PFM strength.*	0.025	1.78
Whether abdominal muscle endurance measured at 8 weeks predicts pelvic floor support and symptoms at 1 year.	(a) Greater abdominal muscle endurance at 8 weeks predicts better pelvic floor support 1 year postpartum.(b) Greater abdominal muscle endurance at 8 weeks predicts fewer symptoms 1 year postpartum.	0.05	1.70
The components of habitus, measured at 1 year that are associated with poor pelvic floor support and symptoms at 1 year.	(a) At 1 year postpartum, greater adiposity, as per cent body fat, and body mass index are associated with worse pelvic floor support.(b) At 1 year postpartum, greater adiposity, as per cent body fat, and body mass index are associated with greater symptoms.	0.05	1.70
Whether waist circumference at 8 weeks postpartum predicts pelvic floor support and symptoms at 1 year.	(a) Greater waist circumference at 8 weeks postpartum predicts worse pelvic floor support 1 year postpartum(b) Greater waist circumference at 8 weeks postpartum predicts greater pelvic floor symptoms 1 year postpartum.	0.05	1.70

*We use an adjusted two-sided significance level of 0.05/2=0.025, reflecting two hypothesis tests from two independent.

†We use an adjusted two-sided significance level of 0.017 to accommodate three separate binomial regression models for statistical interaction terms between each test of muscular fitness and IAP.

IAP, intra-abdominal pressure; MVPA, moderate-to-vigorous physical activity; PFM, pelvic floor muscle strength.

We base our sample size estimate on the proportion of women expected to demonstrate pelvic floor support at or below the hymen (ie, MVD≥0 cm indicating worse support). (Based on pilot data, a higher proportion of women are expected to meet the criteria for symptom burden, our second primary outcome.) Of 10 studies identified at the onset of this research that use the POP-Q to assess vaginal support during the first postpartum year, 7 measured support 6–12 months postpartum^20^ ^27^ ^29^ ^87–90^ (total n=1215); and 4 at 6 weeks to 6 months postpartum^21^ ^22^ ^28^ ^91^ (total n=671). The mean anterior vaginal wall support was always worse (more positive) than the mean posterior vaginal wall or mean apical support. The distribution of stage in studies providing this ranged from 0% to 30%, 26% to 65%, 28% to 59%, and from 0% to 5% for stages I, II and III, respectively.^20–22^ The proportion of women with MVD≥0 ranged from 0% to 41%.^20^ ^22^ ^28^ ^87^ ^88^ ^90^ In a pilot study of Utah women, similar to our expected population (unpublished), 18% had MVD≥0 at 1 year postpartum. We elected to conservatively estimate the proportion of women with MVD≥0 as 15%.

Sample size determinations were performed using PASS 2008. Consistent with Dunnett and Goldsmith,^92^ we did not adjust for multiplicity based on our two primary outcomes, as the inference related to the study aim does not require the simultaneous examination of the two comparisons.

Our predictors are continuous variables. We consider women at the mean for a given predictor as ‘low risk’ and those at the mean plus 1SD as ‘high risk’. Assuming that the frequency of MVD≥0 is 15% in the low-risk group and R^2^ of the predictor regressed on other predictors is 0.5, a sample size of 585 women at 1 year follow-up provides 90% power to detect the minimal OR shown in table 2 for women whose predictor value is 1SD higher than other women whose value is at the mean. (R^2^ is likely to be lower; if so, this sample size provides 90% power to detect even lower odds).

### Study aims and adequacy of sample size for the qualitative project

The aims for the qualitative project are given in box 1.

Box 1 **Study aims for the qualitative project**

1. To describe primiparous Mexican-American and Euro-American women's experiences and cultural knowledge of postpartum pelvic floor support changes.
  - To characterise the ways women perceive and make sense of early changes in pelvic floor support as well as the ways they use language and discourse to construct meaning about those changes in the year after their first delivery.
  - To describe how primipara share experiences and cultural understandings of postpartum pelvic floor support with mothers, partners, sisters and confidantes in their families and social networks.
  - To explore the interplay of women's understandings of early changes in pelvic floor support with sociocultural prescriptions/proscriptions regarding physical activity and any resulting postpartum alterations of activity which they may undertake.
2. To collaboratively develop the toolkit of culturally appropriate resources for women, their families and clinicians; to evaluate it for applicability; and to deploy it on a nationally accessible website.

The planned sample of 30 Euro-American and 30 Mexican-American new mothers is expected to achieve conceptual saturation of the data based on prior ethnographic studies;^93^ ^94^ that is, we expect that no new relevant themes will be identified from successive interviews, no new relevant categories for a subsequent interviewee will be suggested by the data, and ongoing member checks will validate the developing findings.

### Study discontinuation

A participant will be withdrawn from the primiparous cohort if she does not wear the accelerometer at either 2–3 or 5–6 weeks AND does not complete the 8-week study visit.

### Data analysis

Requirements for reporting as outlined by the STROBE^95^ guidelines will be followed for projects 1 and 2 and by CORE_Q^96^ or equivalent for project 3.

#### Quantitative data

The study population will consist of primiparous women who delivered vaginally and were followed longitudinally for 1 year. We will perform descriptive statistics, stratified by ethnicity, to characterise the population and will characterise the trajectories of key variables by plotting longitudinal patterns at the third trimester, and at 8 weeks and 1 year postpartum.

In this cohort study, we cannot remove prevalent cases as it is not feasible to measure pelvic floor support in this large population before pregnancy, though cases of MVD≥0 are rare in nulliparas.^97^ Since we cannot estimate true risk ratios, we will calculate the prevalence ratio for each outcome at 1 year postpartum based on each predictor, as well as model prevalence ratios using modified generalised linear models, such as logistic regression with variance correction by GEE.^98^

Analyses of dichotomous outcomes will begin with a univariate analysis of prevalence ratios and CIs for the primary predictor and potential confounders. Further modelling by multivariable modified logistic regression will adjust potential confounders based on a directed acyclic graph.^99^ ^100^ We will avoid adjusting for variables downstream of a predictor in the causal path.^99^ Effect modification will be considered when cell sizes permit. Models will be checked for adequacy of the model, multicollinearity, influential observations, etc, using standard regression diagnostics. We do not anticipate time-dependent confounders since each hypothesis addresses a specific time relative to delivery. In the final analysis, missing values will be imputed as needed using sequential regression multiple imputation (ICE in Stata or IVEWARE with SAS).^101^

Nominal p values and 95% CIs will be reported. All tests will be two-sided, at the conventional 5% significance level, except as noted. When there are multiple tests to be performed simultaneously, for example, several scenarios or several predictors to be tested in separate models, we will use a proper multiple test correction to adjust for the inflation of type I error. Analysis will be performed in SAS, Stata or R.

#### Qualitative data analysis

After verifying the accuracy of each transcript against the audiotape (and verifying the accuracy and completeness of the English translation, by bilingual/bicultural research team members, in the case of the Spanish language interviews), we will use atlas.TI V.7–8 qualitative analysis software to code the data in the original language of the interview.

The sensitising interviews will be used to refine the interview procedures with the primiparous sample and form the basis of the inductively generated coding dictionary for the study. Mexican-American and European-American women's data will be analysed within each cultural group and then comparatively across groups. Team-based qualitative coding using descriptive and in vivo codes will be used in the first cycle of coding.^102^ Second-cycle coding will identify process and axial codes^103^ helpful for building data-derived concepts useful in comparing the experiences of women within and across the two ethnic group samples.

### Procedures to increase validity/minimise bias

Quantitative bias, a threat to validity, could occur when the same coordinator collects both outcomes and predictors. The anatomic primary outcome is based on the POP-Q evaluation performed at 1 year and not at 8 weeks. At 8 weeks, researchers will collect weight and POP-Q first and will follow a specific manual of operations, including a script. They will terminate the abdominal muscle endurance test based on the script, not based on looking at time taken for the test and will look at time only after terminated. The IAP is already masked, in that the pressure units are calculated later and are not available to the coordinator. At 1 year, a research staff member based in obstetrics/gynaecology, blocked from accessing already entered examination and questionnaire data, will collect those procedures that require vaginal examination: POP-Q, IAP and pelvic muscle strength testing. An exercise science research assistant will measure weight, height, body composition and hand grip strength. Based on the large number of participants, the likelihood is low that researchers will remember the findings they obtained at 8 weeks at the 1-year visit.

To safeguard qualitative validity, we employ a variety of strategies.^86^ Descriptive validity will be addressed by conducting interviews with highly trained researchers with the linguistic and cultural background and women's health expertise to elicit a rich description of the woman's experience. The bilingual/bicultural researchers will shape the research team understanding of nuanced linguistic and cultural elements of the experience. Interpretive validity will be addressed through the team-based data coding and theorising process. An audit trail is built into the process of developing and refining the coding dictionary, with codes linked to memos that explain the evolution of researchers' ideas and theoretical insights.

### Primary study limitations

While a randomised trial design would be the most rigorous to address the research questions for our quantitative aims, it would be unethical to limit physical activity, muscular strengthening, weight loss, etc, after delivery and indeed difficult if not impossible to promote adherence to such interventions, given the varying cultural norms about activity practices after delivery. There are several limitations that are of particular note with our prospective cohort study: (1) generalisability: while we will over-recruit Mexican-American women, our final population will not reflect the population of the USA. By including only primiparous women, we will not be able to draw conclusions about the effects of our study variables on pelvic floor health in multiparous women, although these women would be more likely to already have delivery-related deteriorations in anatomy or function than women recruited during their first pregnancy. (2) We assume that mitigating factors associated with vaginal descent 1 year postpartum could also mitigate end-stage POP decades later. Unfortunately, it is not feasible to conduct the optimal studies to assess the impact of postpartum lifestyle factors on an outcome often seen 20–40 years later. We know that (1) vaginal delivery is the strongest risk factor yet identified for future POP, (2) specific delivery factors are associated with impaired vaginal support both at 1 year and at 5–10 years after delivery, (3) women with postpartum urinary or faecal incontinence are at increased risk for persistent or recurrent bothersome incontinence decades later and (4) women recruited from the community with less vaginal support are those most likely to experience worsening of support over time. While there are no longitudinal studies that test whether women with impaired support postpartum are the same women who develop bothersome POP 3–4 decades later, that other postpartum symptoms are associated with future conditions, and that first delivery has a great impact on future POP are consistent with (though do not prove) the association between postpartum deterioration in vaginal support and future POP. Proving this association requires a 3–4 decade long cohort study; decades of inaction awaiting such results before shifting therapy from symptomatic management later in life to preventative measures after delivery is unwarranted. Further, while young women are unlikely to have end-stage POP, they do have a plethora of symptoms that are understudied; our data can suggest interventions that will improve their quality of life while young. (5) Follow-up: a high withdrawal rate affects the integrity of cohort studies and is a potential limitation for all cohort studies. Historically, our follow-up rates for studies of postpartum women have been high and we have instituted various measures to maximise follow-up in this study.

### Ethics and dissemination

The projects described have been reviewed and approved by the University of Utah and Intermountain Healthcare Institutional Review Boards and have been designated as no more than minimal risk. After participants sign informed consent documents, study personnel will document key elements of informed consent and store the consent document in a locked file cabinet.

To mitigate risks related to confidentiality of data, women are assigned a study identification number. A separate file linking study identification numbers with contact information will be maintained for the duration of the study. Records will be kept in locked filing cabinets until entered on password-protected computers. Questionnaire and data obtained during the examinations will be entered into Research Electronic Data Capture (REDCap), a secure, web-based application designed to support data capture for research studies.^104^ Larger data sets, including IAP and accelerometry data, are stored on BOX, a secure, password-protected online cloud storage and collaboration tool. The Information Security Office at the University of Utah has approved the use of the University’s Box.com installation for storage of HIPAA and Family Educational Rights and Privacy Act (FERPA) data.

Digital audiofiles generated during the qualitative interviews will be uploaded onto the transcription service's protected server. When transcription is complete, a member of the research team will download the transcript onto the password-protected, secured study computer and substitute names with pseudonyms to produce an anonymised transcript. Field notes and any other printed participant materials will be kept in locked files when not in use. The de-identified interview transcripts will be saved as an archive for future research.

Research personnel undergo training and certification in all aspects of the study, ranging from eligibility screening to conducting study procedures. Training and certification are coordinated by study personnel with prequalified expertise. For pelvic floor support, a primary outcome, coordinators are trained in POP-Q examination by (1) didactic sessions, (2) book, article, video and PowerPoint review, (3) attending multiple clinic and operating room sessions in urogynaecology, (4) interactive web-based tools, (5) conducting examinations on trained paid female volunteers, and (6) conducting examinations on willing patients in the urogynaecology clinic under direct faculty supervision. The primary certification standard is to successfully demonstrate during 10 examinations 100% concordance on classification of ‘better’ (MVD<0 cm) versus ‘worse’ (MVD≥0 cm) pelvic support.

We have convened a Safety Committee with representatives from Maternal Fetal Medicine, Kinesiology, Nursing and Physical Therapy, which will meet twice yearly to review a summary of reported adverse events and as needed to review unanticipated problems. Study personnel are instructed to report the following events, as well as any other event that may constitute an adverse event: vaginal irritation, bleeding, pain, tingling, heat or burning related to the examination, emotional distress, wrist irritation related to the accelerometer, more than transient muscle soreness related to strength testing, breach of confidentiality, stigma/social harm (as expressed by the participant) and privacy breach.

We will disseminate results from the primary and secondary aims of the projects, as well as those from ancillary studies related to the projects, in meeting abstracts and in peer-reviewed publications.

To enhance translation of MAP's results into practice, and to provide cultural context for prevention interventions, the results of all three projects will be incorporated into a resource toolkit, together with existing resources, and posted at a nationally accessible website.

## Discussion

We have described the methods for the MAP study that seeks to understand the influence of IAP, physical activity and muscle fitness on pelvic floor support and symptoms and the cultural context in which women experience those changes. The questions raised by these projects address fundamental issues in pelvic floor support. Many of our questions are novel; for example, does moderate-to-vigorous physical activity in the early postpartum period place women at risk for worse pelvic floor support and greater pelvic floor symptoms? Does greater muscle fitness protect the pelvic floor? Others are questions that have persisted unanswered, subject to speculation and controversy; for example, Does elevated IAP predict worse pelvic floor support and greater symptoms? We use novel resources, including a vaginal transducer system developed by a collaboration among our bioengineering, exercise science and urogynaecology researchers, which allow us to measure IAP in real-world settings.

We recognise that the quantitative projects provide mechanistic evidence for developing innovative prevention strategies. However, future advances in preventing PFDs will only be achieved if research efforts are also directed towards understanding the implementation context for such preventive strategies. By also studying the cultural context of early changes in vaginal support experienced by women after childbirth, we will provide vital information needed to direct future preventive efforts.

## Conclusion

Rather than assuming that intrapartum interventions represent the only option for primary prevention of PFDs, we test whether factors during the first postpartum year influence recovery, demonstrated by pelvic floor support and symptoms. Finding relationships between physical activity, muscular fitness, and pelvic floor support and symptoms will provide realistic targets for culturally appropriate disease prevention and pelvic floor health management.
